# Tuberculum meningioma with recovery of glaucoma-like visual field defects after chiasmal decompression: a case report

**DOI:** 10.1186/s12886-024-03332-w

**Published:** 2024-02-14

**Authors:** Kaori Hanai, Masato Hashimoto, Hirohiko Nakamura

**Affiliations:** 1 Department of Ophthalmology, Nakamura Memorial Hospital, S-1, W-14, Chuo-ku, 060-8570 Sapporo, Japan; 2 Department of Neurosurgery, Nakamura Memorial Hospital, S-1, W-14, Chuo-ku, Sapporo, Japan

**Keywords:** Suprasellar tumor, Chiasmal compression, Preperimetric glaucoma, Optical coherence tomography (OCT)

## Abstract

**Background:**

To report a case of tuberculum meningioma with recovery of glaucoma-like visual field defects after chiasmal decompression.

**Case presentation:**

A 39-year-old woman presenting with headache was found to have bilateral arcuate retinal nerve fiber layer (RNFL) thinning on optical coherence tomography (OCT) with a corresponding arcuate scotomas consistent with glaucomatous change. However a suprasellar tumor compressing the anterior chiasm from below was found on magnetic resonance imaging of the brain. After resection of the mass, which was diagnosed as meningothelial meningioma by the pathological examination, the glaucoma-like visual field defects resolved despite the RNFL thinning on the OCT showing no improvement.

**Conclusions:**

Chiasmal compression may mimic glaucoma and produce arcuate scotoma rather than temporal visual field loss. There is a possibility that the development of chiasmal compression somehow converted preperimetric glaucoma into a more advanced form accompanied by visual field defects and that the glaucoma reverted to the preperimetric state after chiasmal decompression.

## Background

Most sellar mass lesions such as tuberculum meningioma or pituitary adenoma produce bitemporal visual field defects and its funduscopic examination typically demonstrates band atrophy of the optic disc, resulting from axonal damage of crossing retinal nerve fibers by direct compression of the chiasm. In contrast, glaucoma is classically characterized by a typical appearance of the optic nerve head including neuroretinal rim thinning and arcuate retinal nerve fiber layer (RNFL) defects or thinning followed by arcuate scotoma. There have been several previous reports of glaucomatous appearance of the optic nerve head in patients with intracranial lesions [1–6]. However, it has remained inconclusive so far, whether the coincidence of tumor and glaucomatous optic neuropathy was incidental or whether it was causally related.

We report a case of tuberculum meningioma with recovery of glaucoma-like visual field changes after chiasmal decompression.

## Case presentation

**Fig. 1:**
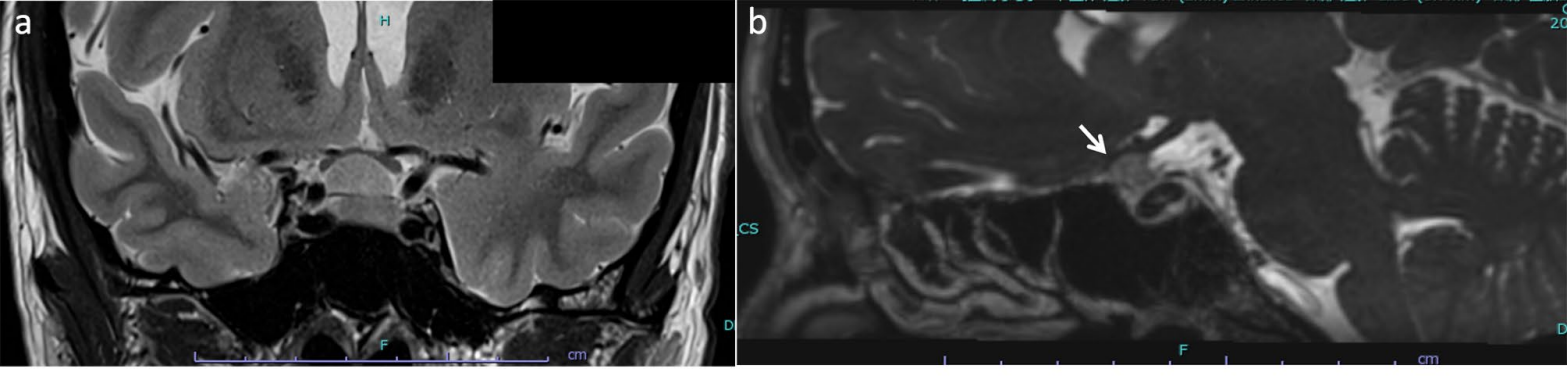
MRI of the brain. **A**: Coronal T2-weighted image demonstrated the suprasellar mass compressing the optic chiasm upwards, especially on the right side. **B**: Post-contrast sagittal image on CISS demonstrated the enhanced mass compressing the anterior chiasm (arrow)

**Fig. 2:**
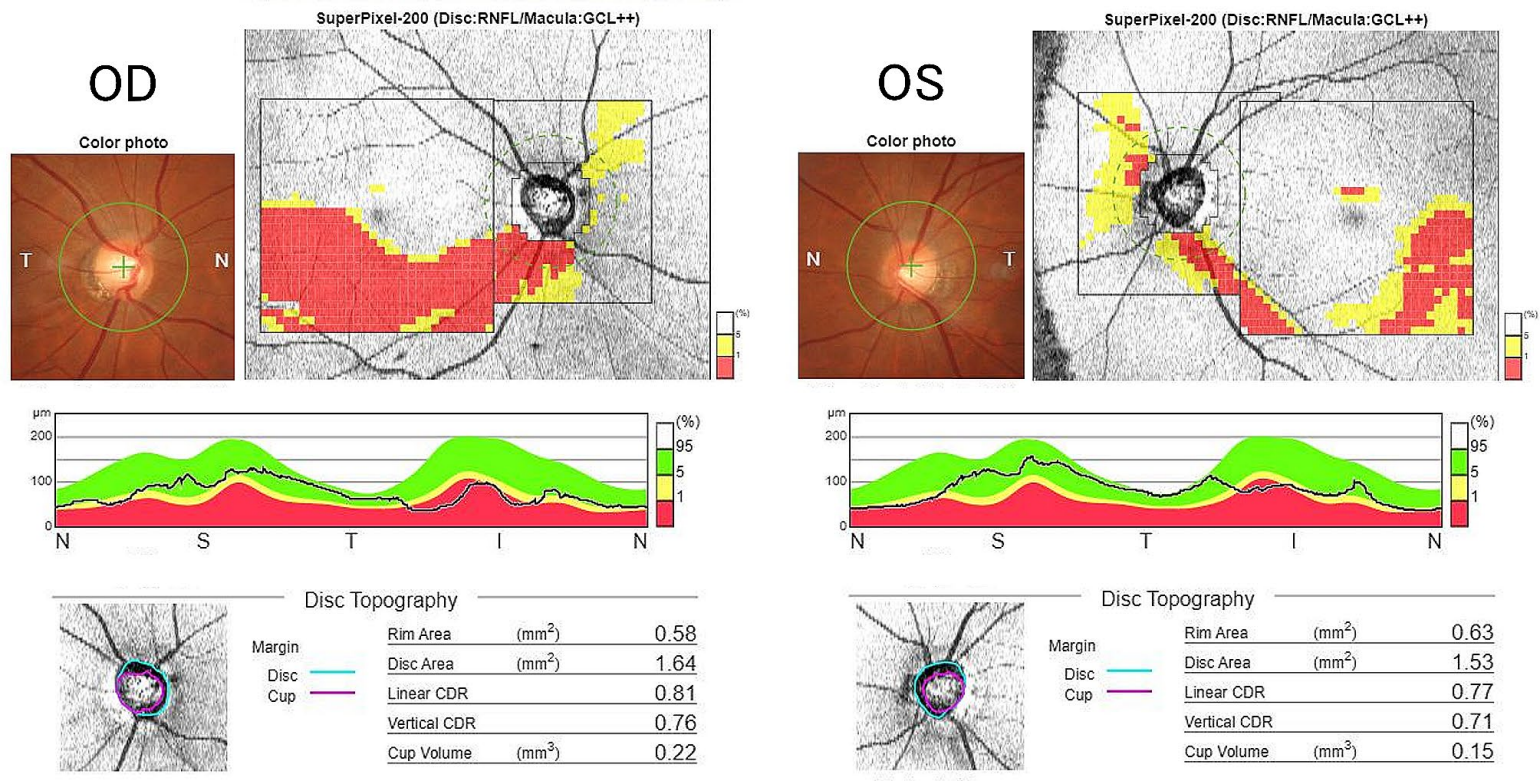
Pre-operative SS-OCT images showed mildly large cups in both optic discs (C/D:0.8 in the right, 0.7 in the left) and inferior arcuate RNFL thinning on the right and mild thinning on the left. Peripapillary RNFL thicknesses demonstrated reduction of inferior segment in the both eyes

**Fig. 3:**
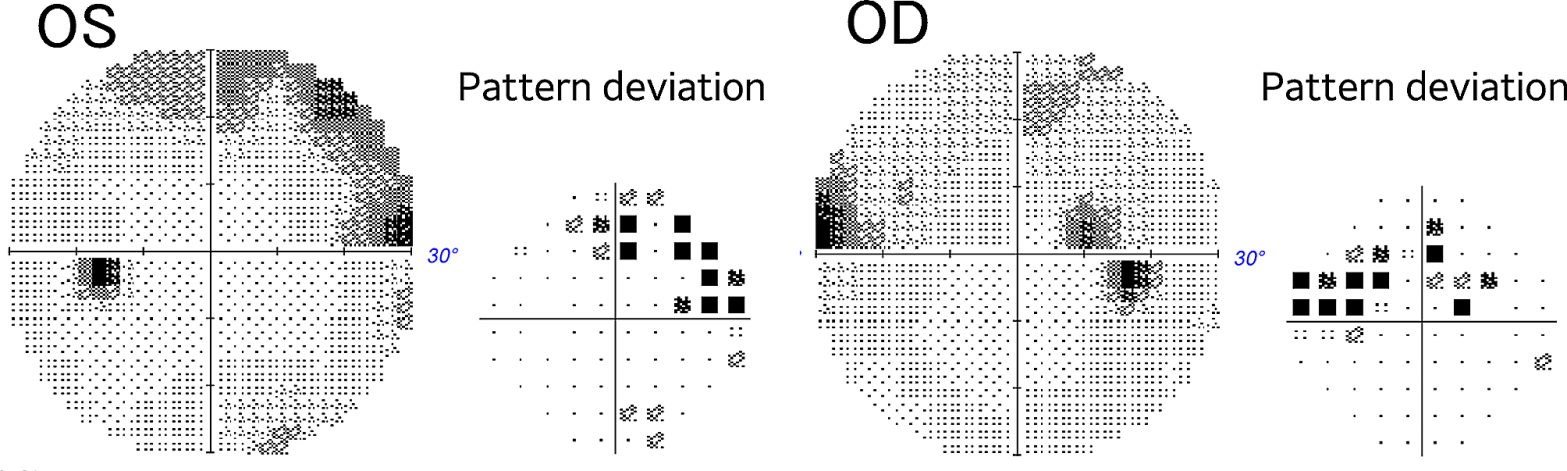
Pre-operative 30 − 2 Humphrey automated visual field examination demonstrating a paracentral upper arcuate scotoma in the both eyes

**Fig. 4:**
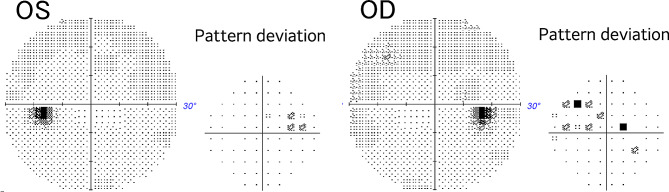
1week post-operative 30 − 2 Humphrey automated visual field examination showing the recovery of the visual field defects

**Fig. 5:**
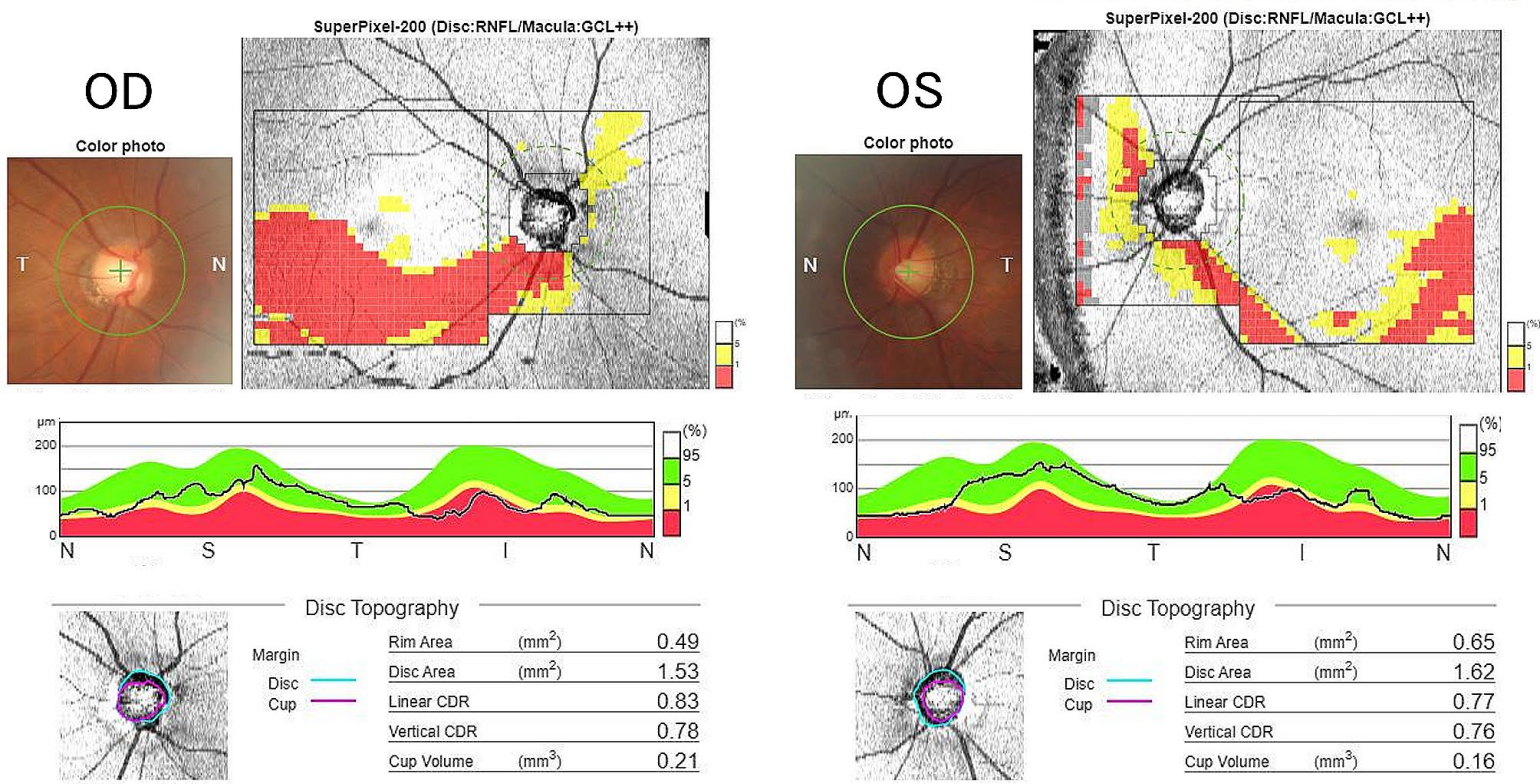
6 months post-operative SS-OCT images showing the same arcuate RNFL thinning as pre-operative images

A 39-year-old woman was referred to the neurosurgery section of our hospital with a chief complaint of headache. Magnetic resonance imaging (MRI) of the brain showed a suprasellar tumor with low signal intensity on T1-weighted image, iso signal intensity on T2-weighted image and post-contrast enhancement on Gadolinium-DTPA. The mass compressed the anterior optic chiasm upwards, especially on the right side (Fig. 1). She had no history of systemic or ophthalmologic disease. She was admitted to the Neurosurgery department and was referred to our Department of Ophthalmology in order to examine her visual function. On examination, visual acuity was 20/20 in each eye (OU) and there was no relative afferent pupillary defect. Intraocular pressure was 14 mmHg OU. Slit-lamp examination showed no abnormalities. Fundus examination showed mildly large optic cups. Using spectral-source optical coherence tomography (SS-OCT), RNFL analysis showed inferior arcuate RNFL thinning on the right and mild thinning on the left (Fig. 2). Automated perimetry revealed upper arcuate scotoma in the right eye larger than the left eye (Fig. 3). The scotomas were compatible with arcuate RNFL thinning and appeared to be consistent with normal tension glaucoma. The patient had never noticed her visual field defects. The suprasellar tumor was wholly resected and the pathological examination resulted in the diagnossis of a meningothelial meningioma. One week after surgery, her visual field defects resolved (Fig. 4). However, the RNFL thinning on OCT was still present regardless the resolution of visual field defects maintained at six months follow up (Fig. 5).

## Discussion and conclusions

The arcuate visual field defects in our patient resolved after the tuberculum meningioma was resected. We have no convincing explanation for this clinical finding. Anatomically, the optic nerves tether the anterior chiasm, thereby preventing it from escaping a growing sellar tumor. On the other hand, the posterior chiasm starts from a higher position whose border is located 4 mm above its anterior border and possibly moves further upwards [7]. Therefore, the anterior chiasm is more vulnerable to the force from below than the posterior chiasm or optic tract. Horton et al. investigated the course of fibers through the primate optic chiasm using different fluorescein tracers in each eye of a monkey [8]. They found that crossing fibers segregate within the anterior core of the chiasm and gradually intermingle with uncrossing fibers posteriorly. Kosmorsky et al. described that higher pressures were recorded in the central portion than in the lateral edge of the chiasm by making measurements in cadavers with simulated pituitary tumors [9]. Consequently, crossing fibers in the core of the anterior chiasm are most vulnerable to suprasellar tumors such as that in our patient, resulting in bitemporal visual field defects, and arcuate scotoma from uncrossing fibers is highly unlikely.

Qu et al. described that large perisellar tumors were associated with a glaucomatous appearance of the optic nerve head [10]. Additionally, they hypothesized that the perisellar tumors located relatively close to the optic nerve may have blocked the free passage of cerebrospinal fluid (CSF) into the optic canal. And then, if the retrobulbar space is collapsed, the trans-lamina cribrosa pressure gradient will be increased, resulting in the similar situation of a normal retrobulbar CSF pressure and an increased intraocular pressure. Some previous clinical studies suggested that a low retrobulbar CSF pressure may play a role in the pathogenesis of normal tension glaucoma [11, 12]. The tuberculum meningioma in our patient was not so large and located only under the anterior chiasm, not blocking the free passage of CSF into the optic canal and orbit.

Preperimetric glaucoma (PPG) is defined as the presence of characteristic glaucomatous changes in the optic disc and increased vulnerability to damage in the RNFL, without the presence of visual field defects detectable with standard automated perimetry test [13, 14]. Shiga et al. described that the inferior region of the retina corresponding to the superior visual field is the most susceptible to progression in PPG as in our patient [15]. As a hypothesis, we speculate that the patient had probably PPG with mild large optic cups and RNFL thinning but no frank scotoma and that physiological conduction block from chiasmal compression due to the tumor might exacerbate the vulnerable arcuate RNFL caused by PPG, resulting in manifestation of arcuate scotomas.

We have not discussed all possibilities about two further causes in this case. On the one hand, it cannot be ruled out completely that compression to the chiasm led to atypical defects. On the other hand, it is cause of the fluctuation of visual fields. The patient might be over-motivated during the second visual field test although rates of false positives (0% OU), false negatives (1% OU) and fixation loss (0/19 OD, 2/22 OS) were low. In any case, we continuously need to follow the visual field changes in this patient in the future.

In conclusion, chiasmal compression causing arcuate glaucoma-like scotoma rather than temporal visual field loss is rare. Possibly, the development of chiasmal compression from the tumor somehow converted preperimetric glaucoma into a more advanced form accompanied by visual field defects and that the glaucoma reverted to the preperimetric state when the tumor compression was relieved. Ophthalmologists should be aware that chiasmal compression may not produce classical bitemporal visual field defect and may mimic normal tension glaucoma.

## Data Availability

All data generated or analyzed during this study are included in this published article.

## Abbreviations

CISS: Constructive interference in the steady state
MRI: Magnetic resonance imaging
PPG: Preperimetric glaucoma
RNFL: Retinal nerve fiber layer
SS-OCT: Spectral-source optical coherence tomography
